# The global burden of *Plasmodium vivax* malaria is obscure and insidious

**DOI:** 10.1371/journal.pmed.1003799

**Published:** 2021-10-07

**Authors:** Katherine E. Battle, J. Kevin Baird

**Affiliations:** 1 Institute for Disease Modeling, Seattle, Washington, United States of America; 2 Eijkman-Oxford Clinical Research Unit, Eijkman Institute of Molecular Biology, Jakarta, Indonesia; 3 Nuffield Department of Medicine, Centre for Tropical Medicine, University of Oxford, Oxford, United Kingdom; Mahidol-Oxford Tropical Medicine Research Unit, THAILAND

## Abstract

J. Kevin Baird and colleagues, examine and discuss the estimated global burden of vivax malaria and it’s biological, clinical, and public health complexity.

**Summary points**

- Estimates of the global burdens of morbidity attributable to acute attacks of *Plasmodium falciparum* malaria typically dwarf those of *Plasmodium vivax*, i.e., hundreds of millions versus tens of millions of cases.
- Global burden estimates take no account of latent and subpatent reservoirs of infections carrying more subtle burdens of illness and death in impoverished settings of malnutrition, coendemic infections, and limited access to quality healthcare. Impacts of chronic malaria on human health may be substantial and are excluded from estimates of burdens of acute malaria.
- Compartments of human infection by *P*. *vivax* beyond vascular patency—vascular subpatency, extravascular subpatency, sexual latency, and hepatic latency—obscure endemic transmission and burdens of infection and illness.
- Long thought to be absent from most of sub-Saharan Africa due to the high prevalence of the Duffy-negative phenotype among residents, recent investigations suggest that widespread reservoirs of transmission may occur across that region.
- Human glucose-6-phosphate dehydrogenase (G6PD) deficiency may also affect susceptibility to infection and directly impact access to effective antirelapse therapy of *P*. *vivax* using 8-aminoquinolines that are dangerous to those patients. Natural polymorphisms of the human cytochrome *P-450 2D6* gene impact parasite susceptibility to primaquine antirelapse therapy at population levels.
- All these factors impose great complexity in considering estimates of burdens of *P*. *vivax* and access to effective mitigation of the harm caused. The conventional diagnostics underpinning epidemiological and clinical understanding of vivax malaria may be inadequate to the biology of this parasite.

## Introduction

The global burden of malaria is often reported as a single value that combines the malarias caused by the 5 species of plasmodia that naturally infect humans [1]. The vast majority of this burden is attributable to *Plasmodium falciparum* and *Plasmodium vivax* malarias, which have only recently begun to be reported separately in the World Malaria Report [2]. The estimated annual burden of *P*. *vivax* malaria (14.3 million [13.7 to 15.0 million]) is an order of magnitude lower than that of *P*. *falciparum* (193.5 million [142.0 to 254.7 million]) [3,4]. Infections with *Plasmodium ovale* or *Plasmodium malariae* are geographically widespread but only rarely prevalent at detectable ranges, whereas the zoonosis caused by *Plasmodium knowlesi* of Southeast Asian macaques occurs only in forested areas of that region. The contributions of these 3 minority malarias to the global burden of acute malaria have not been credibly estimated but are likely to be <5%.

Authoritative estimates of burdens of *P*. *falciparum* and *P*. *vivax* refer specifically to events of clinical illness associated with patent parasitemia [3,4]. The incidence of infection per se is not accounted, despite asymptomatic carriers of infection representing an important and often dominant state of infection with regard to either species [5,6]. Semi-immune older children and adults through much of endemic sub-Saharan Africa, who greatly outnumber vulnerable young children and pregnant women, are very often infected at high rates of prevalence but only rarely suffer attacks of acute malaria [7]. Nonetheless, so-called asymptomatic infections may not be considered benign because chronic malaria carries substantial health consequences [8–10]. This naturally acquired immunity associated with chronic malaria has long been attributed to very high rates of exposure to repeated infections occurring almost exclusively in Africa [11]. However, recent work across the endemic globe demonstrates dominance of asymptomatic, microscopically subpatent infections even with relatively low levels of endemic transmission [12–17]. Estimates of global burdens based on clinical attacks likely miss far broader and more subtle indicators of infection and the harm done in often impoverished communities facing myriad other challenges to good health.

A recent study from Indonesia offers important insights regarding that question applied to *P*. *vivax*. Dini and colleagues reported a retrospective analysis of 37,168 patients diagnosed with *P*. *falciparum* and 22,209 with *P*. *vivax* over a period of nearly 10 years [18]. Whereas a diagnosis of *P*. *falciparum* came with a higher risk of death within 14 days of diagnosis, over the longer term, greater numbers of repeated attacks and hospitalizations among *P*. *vivax* patients came with risk of death that was nearly twice that among patients with *P*. *falciparum*. As expressed by those authors in conclusion, “Whilst the acute management of malaria is paramount to prevent early death, our analysis highlights the importance of preventing recurrent malaria.” Repeated attacks of vivax malaria carried elevated risk of malaria morbidity and mortality by any cause. Renal, circulatory, and cognitive harm is done by chronic malaria infections [19–24]. Harm is not limited to a single attack of acute malaria but logically extends to the varied insults to health repeatedly endured by many residents living under endemic malaria transmission. So-called silent reservoirs of infection may thus carry subtle but important burdens of illness and death not captured in global estimates of acute attacks of malaria.

The asymptomatic, microscopically subpatent reservoir of blood stage *P*. *vivax* has been described [25–28], and the latent reservoir of hepatic hypnozoites has long been known [29–32]. In both instances, however, understanding of their very significant contributions to both on-going transmission and acute attacks is only recent [33–35]. Yet another reservoir may also prove relevant to this epidemiology: infection of the extravascular spaces of the marrow and spleen [36–44]. This pathophysiology has only very recently been understood, and it transforms how *P*. *vivax* may be viewed biologically, clinically, and epidemiologically. Transferrin receptor CD71 occurs only on erythroblasts and the youngest (stage I of V) reticulocytes, and it is required for *P*. *vivax* merozoite invasion of those cells [45,46]. This points to an infection seated in deep hemopoietic tissues where CD71 naturally occurs in great abundance relative to its near absence among cells within vascular sinuses. Moreover, hematopoietic niches of plasmodial asexual schizogony (and gametocytogenesis) may be less sensitive to blood schizontocides like artemisinin [47]. Plasma-derived extracellular vesicles specific to *P*. *vivax* malaria cause up-regulation of *P*. *vivax*-adhesive molecules (ICAM-1) on spleen fibroblasts [48]. This fundamental biology of *P*. *vivax*—tropisms favoring the tissues of marrow and spleen rather than peripheral blood circulation—may often place the infection beyond the reach of conventional diagnosis, upon which global burden estimates are ultimately based. These biological intricacies of *P*. *vivax* malaria are thus important to consider in weighing estimates of its clinical burden and prevalence of infection ranges.

Important host factors further complicate those considerations of burden and countermeasures against them. Overwhelming predominance of Duffy-negative phenotype in most of sub-Saharan Africa has long been considered the basis of the relative paucity of endemic *P*. *vivax* in that region [49]. Nonetheless, travelers to that continent often acquire *P*. *vivax* infection, and recent work demonstrates widespread infection of Duffy-negative residents [50–53]. Two other host factors—glucose-6-phosphate dehydrogenase (G6PDd) mutations and cytochrome P450 isozyme 2D6 (CYP2D6) polymorphisms—may also impact burdens of *P*. *vivax*. While only G6PDd may interfere with parasite development in the host [54–57], both of these factors directly impact successful therapy of latent vivax malaria. The only therapeutic options against hepatic latency of malaria are the 8-aminoquinolines, and these compounds are both invariably toxic to G6PDd patients (causing a threatening acute hemolytic anemia) and appear dependent on CYP2D6 metabolic processing to generate the therapeutically active derivative [58–60]. A study from Brazilian Amazonia described primaquine-induced acute hemolytic anemia as the dominant cause of blood transfusion [61], and some endemic nations have prohibited primaquine therapy for fear of such harm [62]. A survey of CYP2D6 genotypes in endemic Cambodia showed 29% of residents likely to have significantly impaired CYP2D6 activity phenotypes (predicted by genotype) at high risk of primaquine treatment failure [63].

This review considers the estimated global burdens of vivax malaria against this backdrop of great biological, clinical, and public health complexity. Aspiration for the elimination of endemic *P*. *vivax* transmission from much of Asia and the Americas within a few short years [64–66] imposes the necessity of understanding burdens of infection, in addition to those of both direct and indirectly linked morbidity and mortality. Numbers of diagnosed clinical attacks as a measure of direct morbidity very likely represent a minority of infections and their health consequences.

### Vector biology

Human infection by the plasmodia requires the presence of anopheline mosquitoes, and their distributions and abundance bear directly upon global burdens of malaria. *Plasmodium vivax* is known to be transmitted by over 70 *Anopheles* species with diverse bionomics. In a previous review, it was found that all *Anopheles* species that were incriminated to transmit *P*. *falciparum* could also transmit *P*. *vivax* [67]. However, the converse is not true—not all vectors that can transmit *P*. *vivax* can also naturally transmit *P*. *falciparum*. The minimal temperature at which sporogonic development occurs is lower for *P*. *vivax* than for *P*. *falciparum*. That biology, along with hepatic latency, largely explains the reach of endemic *P*. *vivax* transmission into temperate zones where *P*. *falciparum* only rarely occurs [68].

The bionomics of the dominant vector species in the parts of the world where *P*. *vivax* predominates are known to be more diverse than those that transmit *P*. *falciparum* in Africa [69]. The African region was identified to have 7 dominant vector species, most of them being within the *Anopheles gambiae* species complex. In the Asia-Pacific (where more than 80% of the global *P*. *vivax* burden is found), there are 19 described species in at least several species complexes [70,71]. Transmission of *P*. *vivax* by these diverse vectors defines varied endemic malaria ecologies: forest, coastal, plantation, paddy, hillside, and urban to name a few. Bionomic heterogeneity among the dominant vector species in *P*. *vivax*–endemic areas requires equally diverse vector control strategies extending well beyond mass net campaigns and emphasis on acute case management [72–74]. Bed nets are less effective against Asian anophelines because, unlike indoor and late night feeding African vectors, those mosquitoes tend to favor feeding outdoors early in the evening [74–76].

### Parasite biology

Whereas in *P*. *falciparum* infectious gametocytes emerge only after several days of asexual parasitemia, in *P*. *vivax*, the asexual and sexual forms emerge together [77]. In a humanized liver rodent model, *P*. *vivax* gametocytes emerged directly from hepatic schizogony [78]. Transmission to mosquitoes may therefore occur before onset of symptoms, during early illness, and thus before treatment is obtained. Indeed, gametocytogenesis without prior blood schizogony would infer an ability to sustain wholly silent infection and transmission. Compared to *P*. *falciparum*, ordinary levels of parasitemia in uncomplicated acute attacks by *P*. *vivax* are very often an order of magnitude lower. That contrast has been attributed to the indiscriminate invasion of red blood cells of any age by *P*. *falciparum* versus the obligate preference of *P*. *vivax* for reticulocytes. This phenomenon also underpinned the spurrious idea that *P*. *vivax* is inherently unable to cause serious illness [79]. The bulk of harmful infectious biomass of *P*. *vivax* appears to lie beyond diagnostic reach in the extravascular spaces of deep hemopoietic tissues or splenic sinusoids. This biology imposes conspicuous diagnostic challenges, making both microscopic and immunochromatographic antigen detection (by rapid diagnostic tests (RDTs)) inherently less sensitive for infection by *P*. *vivax* relative to *P*. *falciparum*. Numerous studies bear this out [6,14,80]. It is a significant problem compounded by a presumably naturally acquired (or innate in the instance of Duffy negativity) immunity routinely suppressing asexual parasitemias below the limits of practical diagnosis by microscope or RDT [81, 82]. In any given survey of peripheral blood for the plasmodia by these means, the actual prevalence of *P*. *vivax* may be considerably higher than that detected, and perhaps higher still when including infections involving only the extravascular spaces of other tissues. There is an inverse correlation between prevalence of microscopically patent parasitemia as a proportion of all detectable (by PCR) parasitemias, i.e., vascular subpatency among the infected becomes more frequent as prevalence declines [6,83].

Latency in *P*. *vivax* involves dormant stages in hepatocytes called hypnozoites that awaken over the weeks, months, and few years following inoculation by a feeding mosquito. A single such event may seed the liver with any number of hypnozoites, but somewhere between 1 and 12 may be typical. The sporozoites that immediately develop to hepatic schizonts (tachysporozoites) simultaneously launch invasive merozoites into the blood to initiate symptomatic blood schizogony a week or two later. Sporozoites becoming latent hypnozoites (bradysporozoites) awaken to launch those attacks after variable (geographic location- and strain-dependent) intervals [32,84]. Latent infection may persist as long as several years and through a series of independent clinical attacks. This biology logically aligns with the estimated proportion of clinical attacks of vivax malaria originating from hypnozoites rather than recent mosquito bites being about 80% [85]. Untreated latent infections cause most patent attacks, and latency cannot now be detected by any diagnostic means.

Known features of the biology of *P*. *vivax* point to a relatively deep obscurity of presence. The actual prevalence of infection may substantially exceed that which is routinely detected by conventional means, with each of the following biological traits contributing to that character: (1) tropisms for extravascular spaces of deep organs like marrow and spleen; (2) sharply limited asexual multiplication in peripheral circulation; (3) naturally acquired or innate immune suppression of asexual blood stages; and (4) prolonged hepatic latency as a dominant state of infection.

### Human genetic factors

People lacking expression of Duffy factor on the surface of red blood cells, as occurs in most of sub-Saharan Africa, has been linked to the relative scarcity of *P*. *vivax* infection in that region [86]. Several lines of evidence, however, point to endemic low-level transmission of *P*. *vivax* across much of Africa [87–89]. Among Duffy-negative individuals, infections by *P*. *vivax* have been confirmed at African sites [90–94]. Several surveys reported prevalent serological positivity to specific *P*. *vivax* antigens in areas where *P*. *vivax* malaria in patients or cross-sectional surveys of blood is exceedingly rare or unknown [95–97]. Another study in peer review indicates expression of Duffy factor by erythroblasts in marrow despite Duffy-negative genotype and an absence of Duffy factor in circulating red blood cells [98], raising the possibility of endemic subpatent infections of hemopoietic tissues. If so, Duffy negativity may not protect from infection by *P*. *vivax* but would profoundly impact the character and detectability of that infection, i.e., limiting it to extravascular spaces of marrow (excepting transient gametocytemia). Endemic transmission of *P*. *vivax* may occur across Africa but is obscured by this host genetic factor and obligate parasite tropism for extravascular spaces of deep tissues.

G6PDd is the most common human inherited abnormality, affecting more than 400 million people and occurring at an average prevalence of 8% in malaria-endemic countries [99]. Many dozens of distinct single nucleotide polymorphisms (SNPs) are known, each being associated with variably impaired enzymatic function [100]. These variants tend to occur within distinct ethnic/geographic groups and are classified according to level of residual enzymatic activity compared to normal. The WHO classification is most often applied, where most variants are either Class II or III, representing phenotypes of <10% or >10% of normal activity, respectively [101]. One of the most conspicuous physiological distinctions between those classes may be G6PD activity phenotypes across red blood cells of increasing age. Whereas reticulocytes of the archetypical Class III A- variant of G6PDd of Africa exhibit nearly normal G6PD activity (which more sharply declines with red blood cell age compared to normal), those of archetypical Class II Mediterranean variant have almost none [102]. This biochemistry bears on global distributions of *P*. *vivax* and this inherited abnormality.

G6PDd is among the inherited blood disorders believed to have been selected by survival advantages against endemic malaria [54–57,103,104]. As already explained, *P*. *vivax* exhibits profound tropisms of infection and anatomic location anchored upon CD71 as an essential molecule of invasion. Severely impaired G6PD activity in reticulocytes of Class II variants may thus have great impacts on the parasitism of *P*. *vivax* but almost none in the nearly G6PD-normal reticulocytes of Class III variants, e.g., Mahidol variant in Thailand and A- in Africa [105]. In South and Southeast Asia, where most endemic *P*. *vivax* transmission occurs, Class II variants predominate over those of Class III [106], suggesting a selection pressure at work. Populations residing within endemic zones most likely to benefit from 8-aminoquinoline therapies against latent *P*. *vivax* may also be the most likely to suffer harm caused by them. Rational fear of 8-aminoquinoline toxicity protects the hypnozoite reservoir from aggressive exposure to those drugs in clinical and public health practice [106].

Likewise, poor efficacy of 8-aminoquinolines due to natural polymorphisms of CYP2D6 may also bear upon global burdens of *P*. *vivax*. Initial demonstrations of CYP2D-dependent therapeutic activity of primaquine against hepatic plasmodia in murine malaria models has been similarly observed in humans infected by *P*. *vivax* [60,107]. CYP2D6 polymorphisms associated with null or relatively poor enzymatic activity occurred in patients experiencing failure of directly supervised high-dose primaquine therapy against relapse by *P*. *vivax* [59,107]. The most common allele of CYP2D6 in Asian populations is impaired *10. There may be relatively high risk of therapeutic failure of antirelapse therapies in as many as 40% of Asians exposed to risk of *P*. *vivax* infection [63,108].

**Fig 1:**
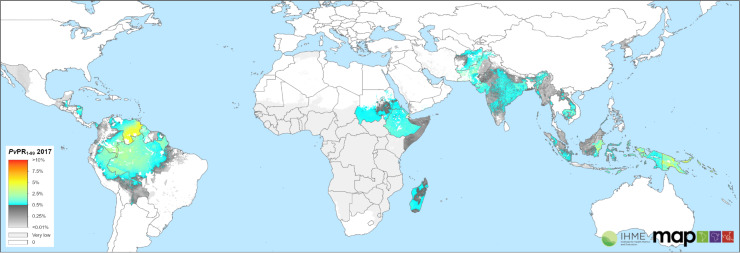
The global prevalence of patent *Plasmodium vivax* in 2017. The mean predicted parasite rate of *P*. *vivax* is in people 1 to 99 years of age based on the analysis from Battle and colleagues is shown on a scale of low prevalence (blue) to high (red) with very low prevalence in light grey and nonendemic regions in white [3]. Global national shapefile obtained from the Malaria Atlas Project (MAP; https://malariaatlas.org/) and available for download through the malariaAtlas R package [109].

Complex human genetic factors exert impacts on the global distribution of burdens of *P*. *vivax* infection, both directly (Duffy negativity and G6PDd) and indirectly through 8-aminoquinoline toxicity (G6PDd) or therapeutic activity (CYP2D6) problems. The direct effects of Duffy negativity may no longer be construed as wholly preventing infection by *P*. *vivax* but profoundly impacting the ability to detect the infection by conventional examinations of peripheral blood. Endemic infection and transmission may occur where Duffy negativity prevails and measures of prevalence of vascular patency seem vanishingly low, as across sub-Saharan Africa (Fig 1).

### Geographic distribution

The dormant liver stage and an ability to develop in its invertebrate host at lower temperatures allow *P*. *vivax* to be the most widely distributed cause of human malaria. *Plasmodium vivax* extends across the endemic tropics, subtropics, and well into temperate climates where the hypnozoite may lie in wait for the seasonal reappearance of feeding anophelines. Keeping in mind the challenges of detection described above, observed prevalence of patent parasitemia values vary greatly across endemic zones, very rarely exceeding 10% prevalence (Fig 1).

A century ago, the global distribution of endemic *P*. *vivax* transmission included most of North America, Europe, and northern Asia and Australia. All of these areas remain receptive to *P*. *vivax* transmission by numerous competent and seasonally abundant vectors. Outbreaks of *P*. *vivax* malaria sporadically occur in these regions, typically in association with migrant human populations [110–113]. The latency of *P*. *vivax*—which is not now possible to diagnose—makes the prevention of imported malaria particularly difficult.

### Populations at risk

**Fig 2:**
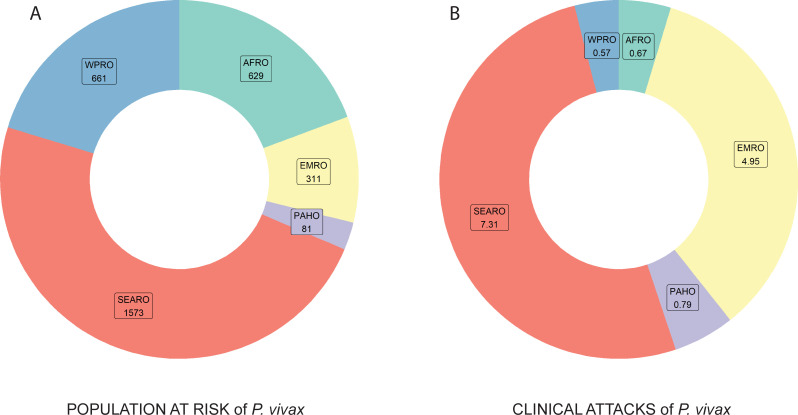
Relative burdens of risk (A) and estimated illness (B) due to *P*. *vivax* malaria among the WHO regions: WPRO is Western Pacific; SEARO is Southeast Asia; EMRO is Eastern Mediterranean; AFRO is Sub-Saharan Africa; and PAHO is the Americas.

Accepting prevalent *P*. *vivax* as evidence of endemic transmission, whether stable or unstable, well over one-third of world’s population (3.3 billion people) were estimated to be living at risk of *P*. *vivax* transmission in 2017 [3]. Of that population, 1.6 billion were in the Southeast Asian WHO region (SEARO), with 80% of the total population in that region being considered at risk. Following SEARO, the Western Pacific (WPRO) and African (AFRO) regions had 661 and 629 million people at risk, respectively. The Eastern Mediterranean Region (EMRO) has nearly half of the population at risk of these regions (311 million), but it disproportionately contributes to the global burden of clinical cases as shown in Fig 2.

The countries with the largest populations at risk are India (SEARO), Pakistan (EMRO), and Ethiopia (AFRO). In much of the *P*. *vivax*–endemic world, urban areas are not considered to be areas of transmission (though there may be incident infections due to relapses or travel). However, in and around the Indian subcontinent, urban areas were not excluded from the populations at risk because of the occurrence of *Anopheles stephensi*, a notoriously competent vector of malaria singularly able to thrive in urban settings [71]. Recent evidence demonstrates that *An*. *stephensi* also occurs on the Horn of Africa, including Ethiopia [113]. The range of this dangerously capable and adaptable vector thus includes a very substantial segment of the population living at risk with stable *P*. *vivax* transmission. Among the predicted 14.3 million cases in 2017, 15% were estimated to have been acquired in urban settings [3]. The near success but ultimate failure to eliminate malaria from India during the 1960s and 1970s has been attributed to the neglect of control in urban areas serving to reseed already cleared rural areas [114]. *Plasmodium vivax* malaria remains predominantly a disease of rural settings; of the 85% of cases occurring beyond urban centers, 64% (7.7 million) cases are estimated to have occurred in rural settings.

### Burdens of infection and disease

**Fig 3:**
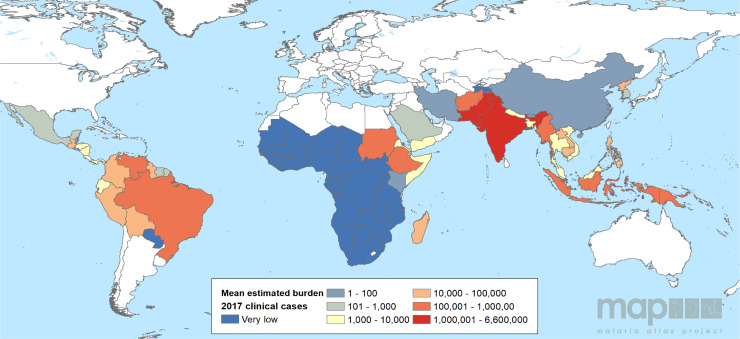
National clinical burdens of *P*. *vivax* malaria. The mean estimated clinical burdens of *P*. *vivax* malaria are shown on a scale of blue (very low burden) to red (high clinical burden) [3]. Global national shapefile obtained from the Malaria Atlas Project (MAP; https://malariaatlas.org/) and available for download through the malariaAtlas R package [109].

Estimates of burden refer to the annual incidence of clinical (symptomatic) infections. The largest burden of these acute attacks occurs on the Indian subcontinent and the Horn of Africa [3], as shown in Fig 3. As discussed above, this metric may narrowly represent broader burdens of morbidity and mortality occurring in connection with chronic infection but more subtly than with a successfully diagnosed and reported event of clinical malaria. Chronic subpatency and latency are examples of this subtlety. While not every individual infected by *P*. *vivax* will experience multiple relapses, it very commonly occurs [30] with “remarkable periodicity,” which varies geographically [84]. The presence of the parasites in the liver is overdispersed—not every infection will lead to the formation of hypnozoites, but those individuals with hypnozoites are more likely to carry at least several more, and, absent specific therapy, will go on to experience multiple relapses within 2 or more years [35]. Infection with *P*. *vivax* in Indonesia, where *P*. *vivax* has a frequent and rapid relapse periodicity, has been shown to have chronic impacts on individuals leading to rehospitalizations with malaria and even early death [18]. In contrast, *P*. *vivax* in India tends to relapse infrequently and at long intervals after infection. Graphic representations of clinical burdens like that of Fig 3 may thus be insensitive to broader health impacts of endemic *P*. *vivax* malaria.

What is true for the burden of clinical disease is also true for infection prevalence. Measured and modeled prevalence estimates like those in Fig 1 refer to infections patent by microscopy or RDT. Low-density subpatent infections are common with *P*. *vivax*, and, as already discussed, subpatency very likely includes infection of extravascular spaces of some deep organs. Standard diagnostic methods are unable to detect vascular or extravascular subpatency. That is also true of the vitally important hypnozoite reservoir. Prevalence of patent infection only narrowly represents broader and probably dominant states of infection, and conventional diagnostics-dependent estimates of burdens of infection are very likely to be minimally representative of true burdens of infection.

Credible vivax malaria diagnostics may require joining the list of human infections reliably diagnosed primarily or solely by serological means. Serological evidence of recent exposure to *P*. *vivax* could prompt therapy without regard to symptoms or conventional diagnostic outcomes. Serological surveys across sub-Saharan Africa, for example, suggested a true prevalence of *P*. *vivax* infection in the range of 11% to 60%, whereas by conventional diagnostics, it appears almost wholly absent [94–96]. This contrast—highly prevalent versus absent—highlights the gravity of diagnostic approach in striving to assess the extent to which *P*. *vivax* infects human communities. Assessments of *P*. *vivax* global burdens of infection will likely require recalibrations based on validated serological diagnostic approaches. Work on those techniques is in progress [115,116].

## Conclusions

**Fig 4:**
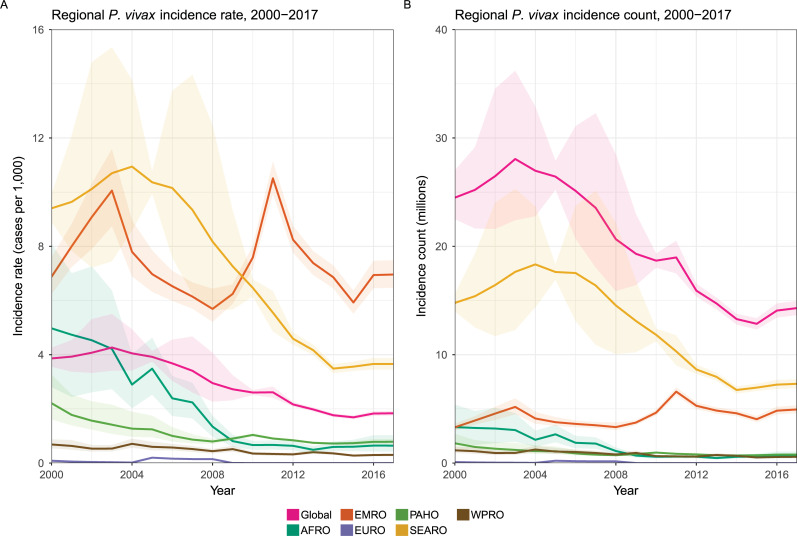
Global and region trends of *P*. *vivax* clinical incidence rate (A) and case counts (B) from 2000 to 2017 [3]. WPRO is Western Pacific; SEARO is Southeast Asia; EMRO is Eastern Mediterranean; AFRO is Sub-Saharan Africa; and PAHO is the Americas.

Endemic *P*. *vivax* transmission occurs across the tropics and reaches into subtropical and temperate climates. Since 2000, the estimated number of patent *P*. *vivax* clinical cases as fallen from 24.5 (22.5 to 27.0 95% CI) million to 14.3 (13.7 to 15.0 95% CI) million in 2017. However, malaria control has seen stagnated progress since 2015, and this holds true for *P*. *vivax* specifically (Fig 4) [3,117]. There has been little change in the estimated burden of acute disease in recent years, and a few countries carry more than 80% of the global case load, i.e., India, Pakistan, and Ethiopia. Addressing case counts alone, however, overemphasizes densely populated endemic countries. Infection prevalence rates are highest in less populated parts of the world, likely driven by environmental suitability as well as high relapse rates, like those seen in Papua New Guinea and the Solomon Islands [83] as examples. Areas in South America, specifically Venezuela where prevalence rates have been increasing in recent years, also exhibit much higher infection rates than those seen in the countries with highest burden (Figs 1 and 3).

Limited blood surveys for *P*. *vivax* malaria and the coarse resolution of routine surveillance data make estimating subnational patterns of prevalence and incidence challenging (Box 1). Existing data and model outputs indicate that *P*. *vivax* is primarily a rural disease affecting populations with limited access to effective treatment. Fine-scale estimates of the distribution of the burden of disease will continue to improve as routine data are made more readily available at the resolution at which they are collected, such as health facility or district. Finer resolution data also have the potential to disaggregate the populations most at risk of infection or clinical disease. Age-specific estimates for *P*. *vivax*, which are important for control planning and commodity forecasting, are currently derived from a model originally developed for *P*. *falciparum* [118] calibrated using data from Papua New Guinea and Indonesia [119]. Updated routine and clinical trial data would greatly increase the temporal and geographic coverage of age-specific data to inform regional *P*. *vivax* malaria age profiles. Robust models to standardize all-age metrics to age-specific results are imperative now that available treatment regimens may be tailored by age. Metrics indicating transmission or endemicity of *P*. *vivax* malaria are most commonly reported for all ages because of the challenges in detecting the parasite [120].

**Box 1. Uncertainties in global malaria burden estimates**

1. Vascular patency is the basis of estimates of global burden of disease, but this is the minority state of infection by *P*. *vivax* in endemic zones where vascular subpatency, extravascular subpatency, sexual latency, and hepatic latency dominate.
2. The parasitemia of *P*. *vivax* malaria is inherently lower than that in *P*. *falciparum* malaria, and it is therefore more often missed in peripheral blood examinations. Vivax malaria may often be considered a fever of unknown origin and treated presumptively.
3. Innate and acquired immunity to *P*. *vivax* suppress or even prevent asexual parasitemia and acute illness, but the host remains infected and infectious. Geographic distributions of infection based on measurements of vascular patency and illness may disregard broad zones or even regions of endemic transmission. Duffy negativity in sub-Saharan Africa, for example, may deeply obscure endemic *P*. *vivax* transmission.
4. The patent acute attack of malaria is the basis of global estimates of disease, but chronic malaria—be it repeated acute attacks borne of mosquitoes or hyponozoites, or persistant vascular or extravascular subpatency—causes more subtle morbidity and mortality that goes unaccounted.
5. Estimates of the global burden of acute patent malaria certainly serve important surveillance goals, but those should not be misconstrued as representing all morbidity and mortality nor the number of infections or geographic distributions of endemic malaria transmission. The hard work of eliminating malaria will require maps of infection/infectiousness from new diagnostic technologies rather than simple vascular patency.
6. Validated serological diagnosis of recent exposure to the plasmodia may prompt treatment and reporting as malaria without regard to vascular patency or illness. That surveillance may yield nearer-to-true prevalence and geographic distributions of infection by these insidious parasites.

The global burden of infection and disease imposed by endemic *P*. *vivax* transmission is obscured by its biology as both an active and latent infection seated in inaccessible tissues. Complex host genetic factors like Duffy factor negativity phenotype deepen that obscurity, and inherited G6PD deficiency may do likewise, i.e., by enhancing tropisms for those tissues. Likewise, due to the singular problem of 8-aminoquinoline toxicity and CYP2D6 dependency for activity against the latent hepatic infections, our ability to attack that important reservoir is deeply impaired. The insidious character of the harm done by *P*. *vivax* further sheltered this parasite from a determined assault upon it—for nearly 60 years, we neglected developing better diagnostics and more effective therapies [121–124]. We thus inherited diagnostics, chemotherapies, and vector control strategies and tactics optimized and validated for an African *P*. *falciparum* problem that are conspicuously inadequate to endemic *P*. *vivax* anywhere. As endemic nations press the elimination agenda by plying those tools, *P*. *falciparum* wanes, but *P*. *vivax* has, unsurprisingly, proven more tenacious [124,125].

The conventional perspective of human malaria as an infection of peripheral blood obscures broader and more subtle burdens of *P*. *vivax*. The microscopic diagnostic standard for over a century (later joined by antigen capture immunochromatography) from finger stick blood specimens defined the presence or absence of infection. This standard remains firmly in place and underpins the global burden estimates described here, which, as a result, can only enumerate certain aspects of *P*. *vivax* burden (i.e., patent blood-stage infection and clinical/classically symptomatic cases). If infection beyond the vascular sinuses indeed dominates *P*. *vivax* biomass in any given human host, we may have to accept the inadequacy of conventional diagnostics to this biology and the epidemiology informed by it. Doing so opens promising avenues of alternative diagnostics like serology. Expanding the scope of burden estimation to consider (i) subpatent infection and (ii) indirect morbidity and mortality is important and firmly on the agenda as and when understanding improves and data allow for it.

## Abbreviations

AFRO: African WHO region
CYP2D6: cytochrome P450 isozyme 2D6
EMRO: Eastern Mediterranean WHO region
G6PD: glucose-6-phosphate dehydrogenase
RDT: rapid diagnostic test
SEARO: Southeast Asian WHO region
SNP: single nucleotide polymorphism
WPRO: Western Pacific WHO region
